# Establishment and validation of a gasdermin signature to evaluate the immune status and direct risk‐group classification in luminal‐B breast cancer

**DOI:** 10.1002/ctm2.614

**Published:** 2021-11-06

**Authors:** Chenxuan Yang, Jiaxiang Liu, Shuangtao Zhao, Jianming Ying, Yueping Liu, Li Ma, Qingyao Shang, Xiangzhi Meng, Kexin Feng, Bo Zheng, Changyuan Guo, Xin Wang, Xiang Wang

**Affiliations:** ^1^ Department of Breast Surgical Oncology National Cancer Center/National Clinical Research Center for Cancer/Cancer Hospital Chinese Academy of Medical Sciences and Peking Union Medical College Beijing China; ^2^ Department of Thoracic Surgery Beijing Tuberculosis and Thoracic Tumor Research Institute/Beijing Chest Hospital, Capital Medical University Beijing China; ^3^ Department of Pathology National Cancer Center/National Clinical Research Center for Cancer/Cancer Hospital Chinese Academy of Medical Sciences and Peking Union Medical College Beijing China; ^4^ Department of Pathology The Fourth Hospital of Hebei Medical University Hebei China; ^5^ Breast Center The Fourth Hospital of Hebei Medical University Hebei China

Dear Editor,

We developed a gasdermin (GSDM) signature score to evaluate immune status and predict outcomes for luminal‐B breast cancer (BRCA). BRCA has become the most commonly diagnosed cancer (11.7%) and is the leading cause of cancer‐related mortality in women.^1^ Various studies proposed models for prognosis prediction and subtype classification in BRCA,^2^ yet more precise models are needed. GSDMs regulate pyroptosis, an inflammatory form of cell death,^3^, ^4^, ^5^ and is associated with tumour progression,^6^, ^7^ but an integrative study of different GSDMs in BRCA is still lacking. The current study explores the role of GSDMs in BRCA by interrogating a Chinese patient cohort from National Cancer Center (NCC) and The Cancer Genome Atlas (TCGA) dataset. The study design is shown in Figure S1. We first studied the differential regulation of GSDMs between BRCA and normal tissues using the multi‐omics data from the TCGA. Different subtypes of BRCA exhibited different GSDM mutations (Figure S2A), but the luminal‐B subtype had the most diversity (Figure 1A). We next analysed the transcriptional changes of GSDMs and found that GSDMA, GSDMC and GSDMD were up‐regulated in cancer compared with normal tissues, while the contrary was true for GSDMB and GSDME (Figure 1B). Correlation of their mRNA expressions were also analysed (Figure S2B). The protein levels of GSDMs were detected by immunochemical staining and quantified, which also revealed aberrant expression in cancer (Figure 1C,D).

**FIGURE 1:**
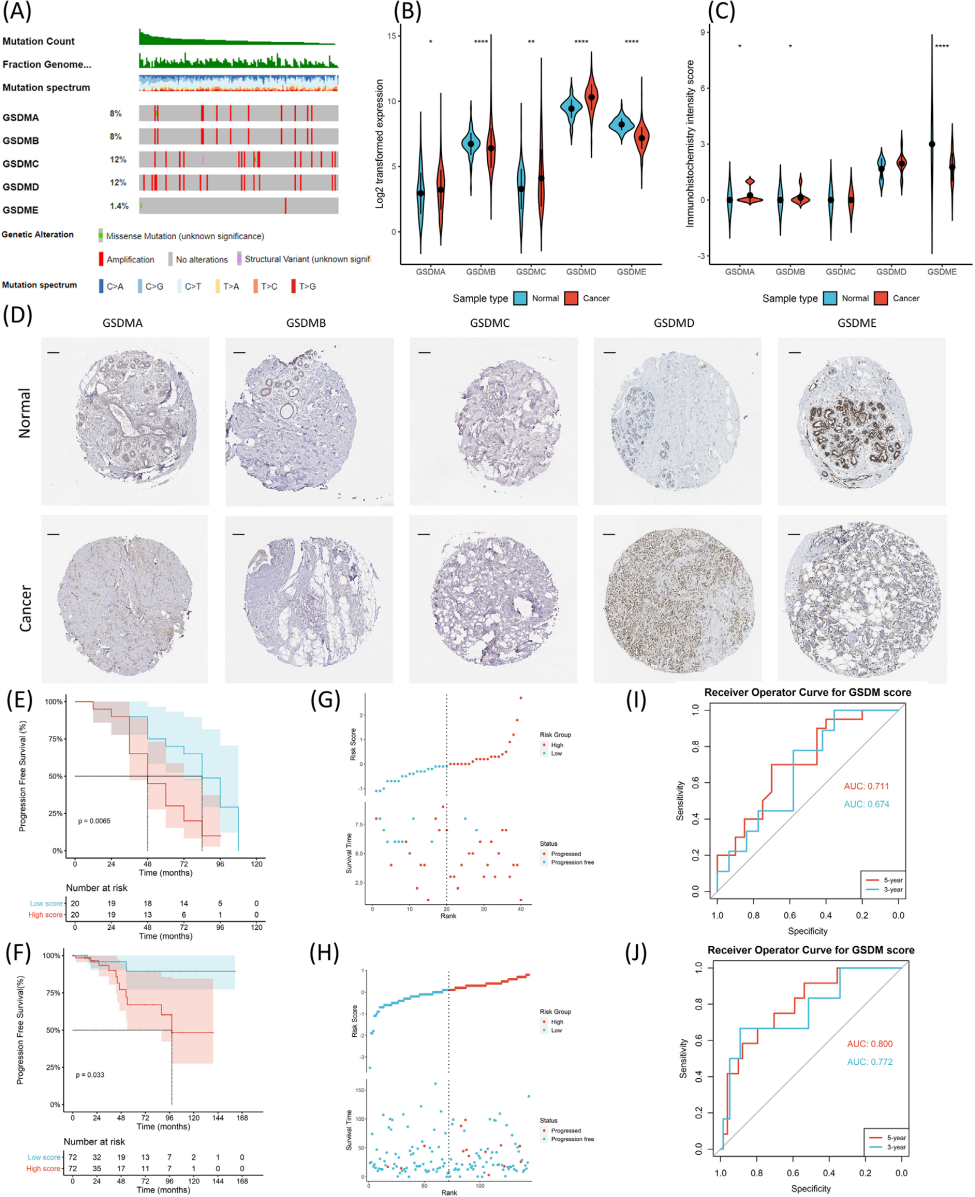
(A) Oncoprint plot of gasdermin (GSDM) mutations in luminal‐B breast cancer (BRCA) from the The Cancer Genome Atlas (TCGA) cohort (*n* = 144). (B) Log_2_ transformed expression of GSDMs in tumours (*n* = 981) compared with normal tissues (*n* = 114) (GSDMA: *p* = 0.0415, GSDMB: *p* < 0.0001, GSDMC: *p* = 0.0034, GSDMD: *p* < 0.0001, GSDME: *p* < 0.0001). (C) Quantifications of the immunohistochemical (IHC) staining (GSDMA: *p* = 0.011, GSDMB: *p* = 0.044, GSDME: *p* < 0.0001). (D) Representative images of GSDM IHC staining in primary resected tumours and normal tissues. Scale bars = 1 μm. (E) Kaplan–Meier plot of progression‐free survival (PFS) in high GSDM score group (*n* = 20) compared with low GSDM score group (*n* = 20, *p* = 0.0065) in the National Cancer Center (NCC) cohort. (F) Kaplan–Meier plot of PFS in high GSDM score group (*n* = 72) compared with low GSDM score group (*n* = 72, *p* = 0.033) in the TCGA cohort. (G) Risk plot depicting PFS status and GSDM score distribution in the NCC cohort. (H) Risk plot depicting PFS status and GSDM score distribution in the TCGA cohort. (I) Receiver operator curve (ROC) curve and area under curve (AUC) for the 3‐year and 5‐year progression rate predicted by the GSDM score (3‐year AUC = 0.674, 5‐year AUC = 0.711) in the NCC cohort. (J) ROC curve for the 3‐year and 5‐year progression rate predicted by the GSDM score (3‐year AUC = 0.722, 5‐year AUC = 0.800) in the TCGA cohort. The Mann–Whitney *U* test was used to compare expressions and IHC intensity scores between tumours and normal tissues. The Log‐rank test was used to compare survival probabilities between different groups. n.s., not significant. *p*‐values: *, <0.05; **, <0.01; ***, <0.001

Since the luminal‐B subtype had the most significant genetical changes in GSDMs, we aimed to investigate the impact of GSDMs on outcomes of this subtype. The Cox regression model was performed to evaluate each GSDM in the NCC cohort. Univariate and adjusted‐multivariate analyses revealed GSDMB, GSDMC and GSDMD as independent prognostic factors (Table S1). We used the multivariate model to construct a GSDM signature score, which was prognostic independent of other clinical factors in both cohorts (Table S2 and S3). Next, we stratified the patients into the high and low score groups using the median as a cutoff. The two groups had comparable clinical features (Table S4 and S5), while the high score group showed significantly worsened outcomes (Figure 1E‐1H, Figure S3A,C). The receiver operator curve (ROC) revealed excellent prediction power of the GSDM signature score (Figure 1I,J). The exact GSDM expressions in each group are shown in Figure S3B,D.

To understand the biological differences, we explored the differentially expressed genes (DEGs) between the high and low score groups using edgeR and limma packages (Figure S4A,B). The representative pathways and molecular functions were then analysed using Gene Set Enrichment Analysis (GSEA) algorithm. The high score group was enriched for cell adhesion and DNA‐binding transcription activation which were associated with tumour invasiveness (Figure 2A). The low score group was enriched for immune and defense responses (Figure 2A). Gene Set Variation Analysis (GSVA) analysis confirmed that the low score group had significantly higher enrichment in interferon responses, yet the high score group was significantly enriched for transforming growth factor‐β (TGF‐β) signaling (Figure 2B). Transcription factor (TF) and kinase enrichment analysis revealed key regulators of the DEGs (Figure 2C,D). We next constructed a protein‐protein interaction (PPI) network to summarise the physical relationships between the DEGs coded proteins (Figure 2E). Seven central clusters were derived by MCODE analysis as the hub genes (Figure S4C). Enrichment analysis for the 31 hub genes was conducted based on their interacting targets, and the enriched terms were largely in line with the previous analysis based on all DEGs (Figure 2F), suggesting that the hub genes are highly representative. To identify potential small chemicals and drugs targeting the hub genes, we also investigated thedrug‐gene interactions and listed the top interacting drugs (Figure S4D,E).

**FIGURE 2:**
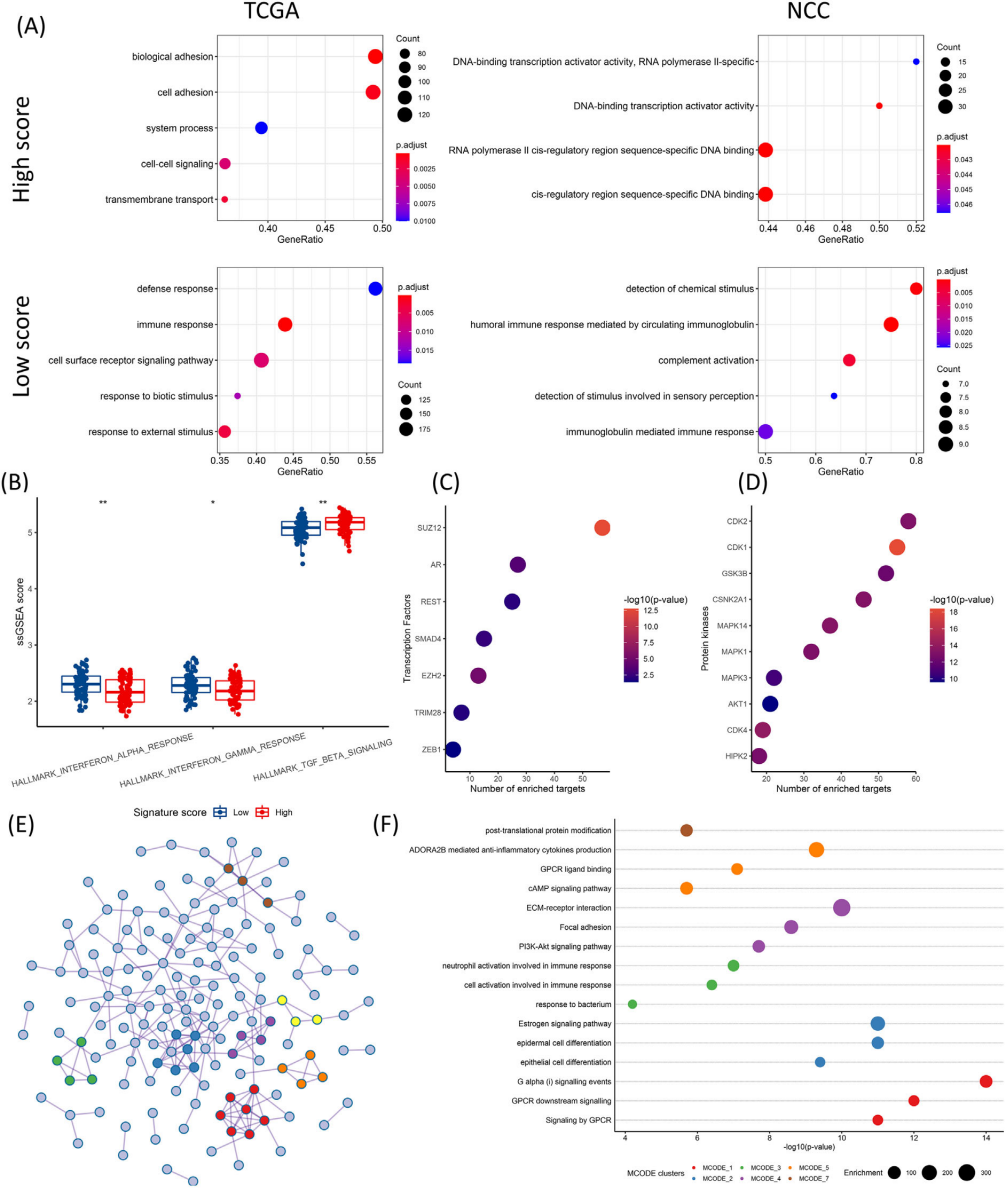
(A) GSEA significantly enriched terms. Top panel: high score group; bottom panel: low score group; left panel: The Cancer Genome Atlas (TCGA); right panel: NCC. (B) GSVA analysis of hallmark gene sets between high and low score groups (interferon‐α: *p* = 0.0013, interferon‐γ: *p* = 0.0199, TGF‐β: *p* = 0.0019). (C) Top enriched transcription factors (*n* = 7) from the differentially expressed genes (DEGs) analysed by X2K. (D) Top enriched protein kinases (*n* = 10) from the DEGs analysed by X2K. (E) Protein‐protein interaction (PPI) network of pooled DEGs, colours depict MCODE clusters. (F) Enrichment terms of different MCODE clusters. The Mann–Whitney *U* test was used to compare GSVA scores between different groups. n.s., not significant. *p*‐values: *, < 0.05; **, < 0.01; ***, < 0.001

GSDMs could modulate immune microenvironment through the release of cytokines and inflammatory contents.^4^ We therefore aimed to study the immunological behaviours of BRCA in high and low score groups. Three sets of immune‐related genes (immunoinhibitors, immunostimulators, and major histocompatibility complex (MHC) molecules) were compared (Figure 3A,B, Figure S5A,C). The majority of immune‐related genes were negatively correlated with the GSDM score, suggesting active immune responses in the low score group. Besides the immune‐related genes, we also evaluated the immune cell components within the tumour tissue. The immune score and stromal score were calculated by ESTIMATE algorithm. The GSDM score was significantly correlated with lower immune cell abundance but not the stromal cells (Figure 3C‐F). We used CIBERSORTx to further calculate the lymphocyte abundance and found that the TCGA cohort showed significantly increased cytotoxic CD8 T cells, regulatory T cells and monocytes in the low score group (Figure 3H, Figure S5D). Similarly, the low score group in the NCC cohort also had significantly increased cytotoxic CD8 T cells (Figure 3G, Figure S5B).

**FIGURE 3:**
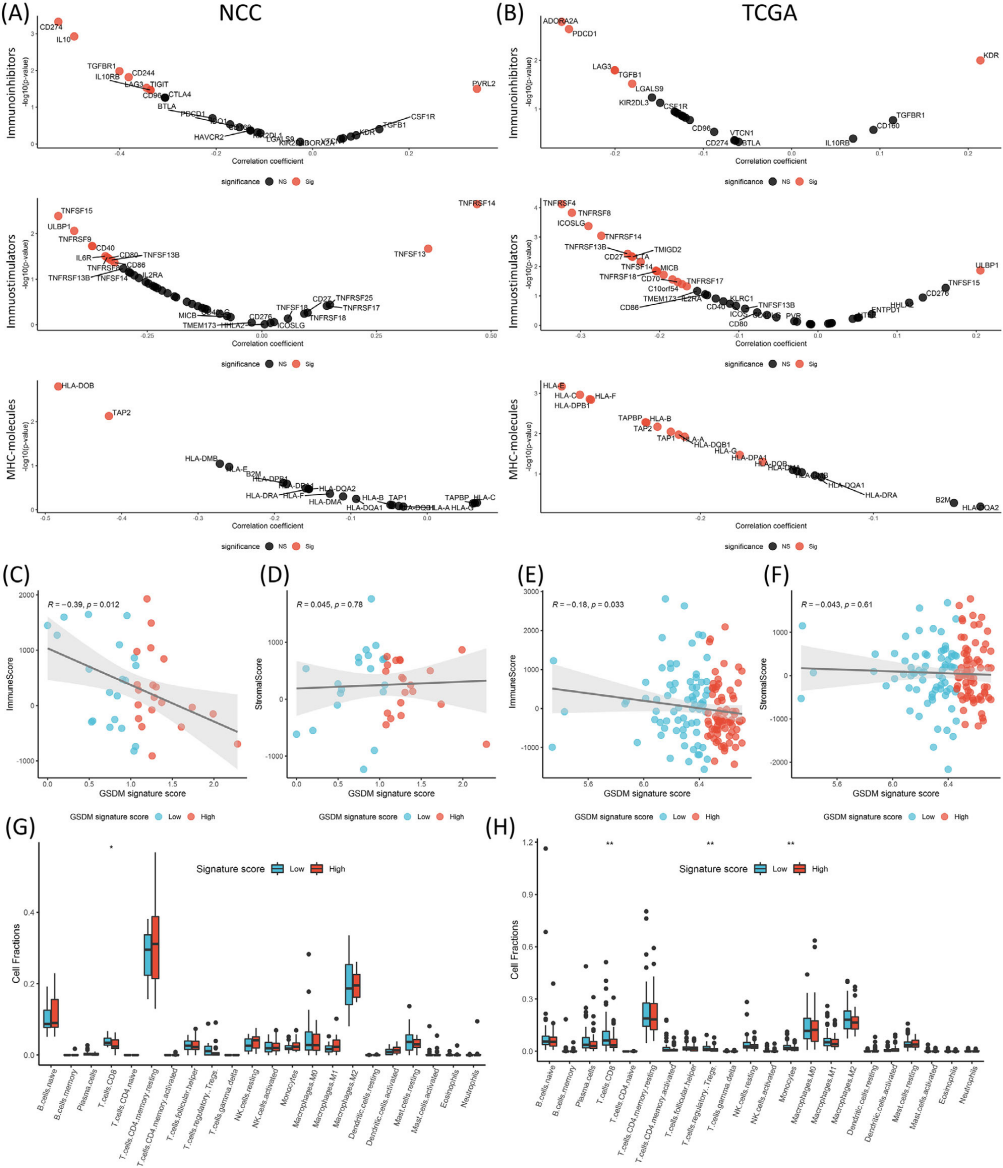
(A) Correlation of immune‐related genes with the gasdermin (GSDM) score in the NCC cohort (from top to bottom: immunoinhibitors, immunostimulators, MHC molecules). (B) Correlation of immune‐related genes with the GSDM score in the The Cancer Genome Atlas (TCGA) cohort. (C) Correlation between the ImmuneScore and the GSDM signature score (*R* = −0.39, *p* = 0.012) in the NCC cohort. (D) Correlation between the StromalScore and the GSDM signature score (*R* = 0.0045, *p* = 0.78) in the NCC cohort. (E) Correlation between the ImmuneScore and the GSDM signature score (*R* = −0.18, *p* = 0.033) in the TCGA cohort. (F) Correlation between the StromalScore and the GSDM signature score (*R* = −0.043, *p* = 0.61) in the TCGA cohort. (G) Tumour infiltrating lymphocytes analysed by CIBERSORTx between high and low score groups (NCC cohort). (H) Tumour infiltrating lymphocytes analysed by CIBERSORTx between high and low score groups (TCGA cohort). The Spearman's rank correlation coefficient was calculated to analyse correlations between different variables. The Mann–Whitney *U* test was used to compare cell fractions between different groups. n.s., not significant. *p*‐values: *, < 0.05; **, < 0.01; ***, < 0.001

To determine the driver mutations regulating the different biological behaviours between the two groups, we analysed the somatic mutations using the whole exosome sequencing data from the TCGA cohort. The high score group had 84.72% of patients carrying somatic mutations, while the low score group had 73.61% (Figure 4A,B). Interestingly, the high score group had a significantly higher mutation rate in GATA‐Binding Protein 3 (GATA3), mainly in the zinc finger (ZnFn) segments (Figure 4C). Most of the GATA3 mutations were frame shift insertions or deletions. Apart from these mutations, the two groups had comparable tumour mutation burden (Figure 4D). The co‐occurrence of somatic mutations in the high and low score groups was determined (Figure 4E,F).

**FIGURE 4:**
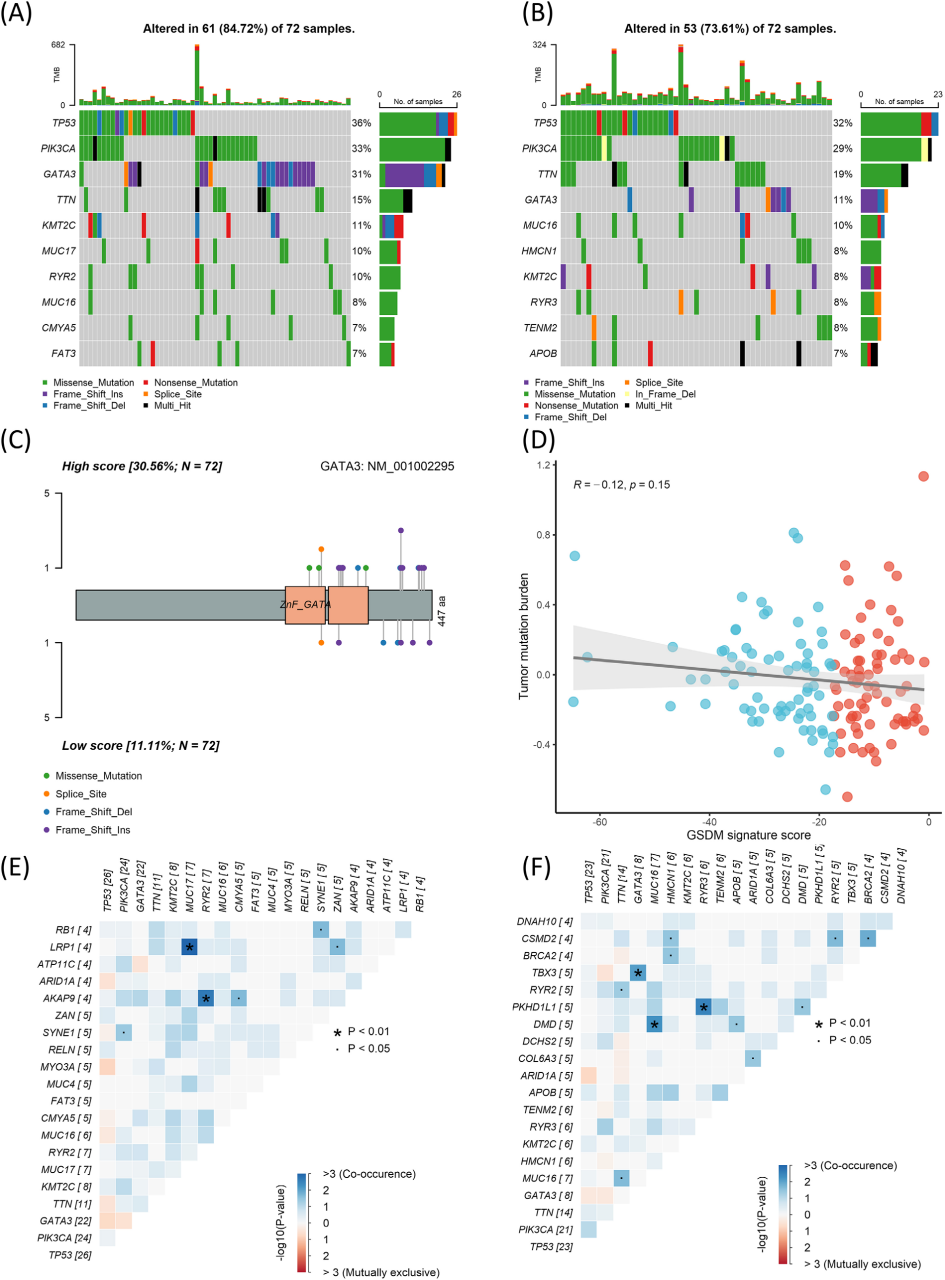
(A) Oncoplot of top mutated genes in the high score group. (B) Oncoplot of top mutated genes in the low score group. (C) Protein structure of GATA3 and the mutation sites in high and low score groups. Top: high score; bottom: low score. (D) Correlation between the tumour mutation burden and the gasdermin (GSDM) signature score (*R* = ‐0.12, *p* = 0.15). (E) Correlation plot of top co‐occurring mutations in the high score group. (F) Correlation plot of top co‐occurring mutations in the low score group. The Spearman's rank correlation coefficient was calculated to analyse correlations between different variables. n.s., not significant. *p*‐values: *, < 0.05; **, < 0.01; ***, < 0.001

The immune checkpoint inhibitors (ICIs) have revolutionized the treatment for various tumours, yet their applications are limited in BRCA and require further understandings.^8^ Different forms of cell death have a major impact on the anti‐tumour immunity and the responses to ICIs. Pyroptosis is one of such ‘immunogenic cell deaths’ which can dramatically alter the tumour immunological landscape.^4^, ^9^ In the current study, we established a GSDM signature score for patient risk classification and presented evidence of differentially regulated intrinsic cellular processes as well as tumour immune status in patients with different GSDM expression profiles. Our findings shed light on harnessing pyroptosis in enhancing anti‐tumour immunity and developing immunotherapies.

## CONFLICT OF INTEREST

The authors declare that they have no competing interests.

## FUNDING INFORMATION

National Natural Science Foundation of China, Grant Number: 82072097; National Key Research and Development Project, Grant Number: 2019YFE0110000; Clinical and Translational Medicine Research Foundation of Chinese Academy of Medical Sciences, Grant Numbers: 2020‐I2M‐C&T‐B‐069, 2017‐I2M‐3‐004; Non‐profit Central Research Institute Fund of Chinese Academy of Medical Sciences, Grant Numbers: 2018PT32013, 2017PT32001 and 2016ZX310178; Beijing Hope Run Special Fund of Cancer Foundation of China, Grant Numbers: LC2017B15 and LC2020A18.

## Supporting information



Supporting Information
