# Supplementary material for: Establishment and validation of a gasdermin signature to evaluate the immune status and direct risk‐group classification in luminal‐B breast cancer
Source: Clin Transl Med. 2021 Nov 6;11(11):e614. doi: 10.1002/ctm2.614 (PMC8571949; doi:10.1002/ctm2.614)
Supplement: Supplementary file 1 — Supporting Information [file CTM2-11-e614-s001.docx]

| Univariate Cox Proportional Hazard Regression |  |  |  |  |
| --- | --- | --- | --- | --- |
| Prognostic factors | HR | P-value | Lower 95% CI | Higher 95% CI |
| GSDMA | 1.4156 | 0.0631 | 0.9811 | 2.0425 |
| GSDMB | 1.0114 | 0.0091 | 1.0028 | 1.0201 |
| GSDMC | 1.0704 | 0.0271 | 1.0077 | 1.137 |
| GSDMD | 0.9798 | 0.1208 | 0.9549 | 1.0054 |
| GSDME | 1.0033 | 0.9182 | 0.9419 | 1.0687 |
| GSDM signature score | 2.7156 | 0.0003 | 1.5757 | 4.6801 |
| Adjusted Multivariate Cox Proportional Hazard Regression |  |  |  |  |
| Prognostic factors | HR | P-value | Lower 95% CI | Higher 95% CI |
| GSDMB | 1.0097 | 0.0297 | 1.001 | 1.0185 |
| GSDMC | 1.0737 | 0.039 | 1.0036 | 1.1487 |
| GSDMD | 0.9742 | 0.0437 | 0.9498 | 0.9993 |

Supplementary Table 1, Cox proportional hazard regression analysis of different GSDMs and the GSDM signature score; HR: hazard ratio; CI: confidence interval.

| Univariate Cox Proportional Hazard Regression |  |  |  |  |
| --- | --- | --- | --- | --- |
| Risk factors | HR | p-value | Lower 95% CI | Higher 95% CI |
| Age | 0.9981 | 0.9364 | 0.9518 | 1.0466 |
| Meuopause |  |  |  |  |
| Pre | 1.0000 |  |  |  |
| Post | 1.7718 | 0.1371 | 0.8335 | 3.7662 |
| History of malignancy |  |  |  |  |
| No | 1.0000 |  |  |  |
| Yes | 1.4512 | 0.5515 | 0.4261 | 4.9427 |
| Tumorsize | 1.4754 | 0.0868 | 0.9453 | 2.3028 |
| Ki.67 | 1.0569 | 0.0210 | 1.0084 | 1.1078 |
| Endocrinotherapy |  |  |  |  |
| No | 1.0000 |  |  |  |
| Yes | 0.8111 | 0.7331 | 0.2436 | 2.7015 |
| Chemotherapy |  |  |  |  |
| No | 1.0000 |  |  |  |
| Yes | 3.6647 | 0.0044 | 1.5002 | 8.9521 |
| Radiotherapy |  |  |  |  |
| No | 1.0000 |  |  |  |
| Yes | 0.3444 | 0.0529 | 0.1171 | 1.0133 |
| GSDM signature score | 2.7156 | 0.0003 | 1.5757 | 4.6801 |
| Adjusted Multivariate Cox Proportional Hazard Regression |  |  |  |  |
| Risk factors | HR | p.value | Lower 95% CI | Higher 95% CI |
| Age | 1.0259 | 0.3720 | 0.9699 | 1.0852 |
| Tumorsize | 1.0019 | 0.9945 | 0.5789 | 1.7339 |
| Chemotherapy | 2.7195 | 0.0412 | 1.0410 | 7.1045 |
| ki.67 | 1.0282 | 0.4476 | 0.9570 | 1.1048 |
| GSDM signature score | 1.9800 | 0.0404 | 1.0304 | 3.8047 |

Supplementary Table 2, Cox proportional hazard regression analysis of clinical status and the GSDM signature score in the NCC cohort; HR: hazard ratio; CI: confidence interval.

| Univariate Cox Proportional Hazard Regression |  |  |  |  |
| --- | --- | --- | --- | --- |
| Risk factors | HR | p-value | Lower 95% CI | Higher 95% CI |
| Age | 0.9844 | 0.4910 | 0.9412 | 1.0295 |
| Meuopause |  |  |  |  |
| Pre | 1.0000 |  |  |  |
| Post | 0.6861 | 0.5262 | 0.2140 | 2.1995 |
| AJCC stage (T) |  |  |  |  |
| T1 | 1.0000 |  |  |  |
| T2 | 2.7360 | 0.1929 | 0.6013 | 12.4492 |
| T3 | 1.1968 | 0.8838 | 0.1077 | 13.3037 |
| AJCC stage (N) |  |  |  |  |
| N0 | 1.0000 |  |  |  |
| N1 | 1.9453 | 0.3636 | 0.4629 | 8.1746 |
| N2 | 4.0145 | 0.0578 | 0.9552 | 16.8717 |
| AJCC stage (M) |  |  |  |  |
| M0 | 1.0000 |  |  |  |
| MX | 3.6011 | 0.0547 | 0.9745 | 13.3070 |
| GSDM signature score | 3.6559 | 0.0469 | 1.0178 | 13.1316 |

Supplementary Table 3, Cox proportional hazard regression analysis of clinical status and the GSDM signature score in the TCGA cohort; HR: hazard ratio; CI: confidence interval.

|  | Low score group (n=20) | High score group (n=20) | P-value |
| --- | --- | --- | --- |
| Age of diagnosis | 50.5 [45.8;54.5] | 53.5 [41.8;58.0] | 0.839 |
| Menopause status: |  |  | 0.747 |
| Pre | 13 (65.0%) | 11 (55.0%) |  |
| Post | 7 (35.0%) | 9 (45.0%) |  |
| History of malignancy |  |  | 0.229 |
| No | 18 (100%) | 15 (83.3%) |  |
| Yes | 0 (0.00%) | 3 (16.7%) |  |
| Tumor size (cm) | 2.06 (0.86) | 2.36 (0.84) | 0.272 |
| Ki.67 index | 21.4 (5.34) | 22.6 (7.57) | 0.535 |
| Endocrinotherapy: |  |  | 1 |
| No | 2 (10.0%) | 2 (10.0%) |  |
| Yes | 18 (90.0%) | 18 (90.0%) |  |
| Chemotherapy: |  |  | 0.751 |
| No | 10 (50.0%) | 8 (40.0%) |  |
| Yes | 10 (50.0%) | 12 (60.0%) |  |
| Radiotherapy: |  |  | 0.695 |
| No | 15 (75.0%) | 17 (85.0%) |  |
| Yes | 5 (25.0%) | 3 (15.0%) |  |

Supplementary Table 4, Clinical demographic status of high and low score groups in the NCC cohort; Chi-square test/Fisher’s exact test or Wilcoxon rank-sum test was used depending on the types of variables. Brackets indicate 25% and 75% quartiles.

|  | Low N=72 | High N=72 | p-value |
| --- | --- | --- | --- |
| Age of diagnosis | 59.5 [49.8;66.2] | 56.0 [48.0;62.2] | 0.212 |
| Menopause status |  |  | 0.948 |
| Other/Unknown | 6 (8.33%) | 7 (9.72%) |  |
| Post | 49 (68.1%) | 49 (68.1%) |  |
| Pre | 17 (23.6%) | 16 (22.2%) |  |
| History of malignancy |  |  | 1.000 |
| No | 71 (98.6%) | 70 (97.2%) |  |
| Yes | 1 (1.39%) | 2 (2.78%) |  |
| AJCC stage (T) |  |  | 0.196 |
| T1 | 17 (23.6%) | 12 (16.7%) |  |
| T2 | 44 (61.1%) | 49 (68.1%) |  |
| T3 | 6 (8.33%) | 10 (13.9%) |  |
| T4 | 5 (6.94%) | 1 (1.39%) |  |
| AJCC stage (N) |  |  | 0.137 |
| N0 | 29 (40.3%) | 30 (41.7%) |  |
| N1 | 25 (34.7%) | 30 (41.7%) |  |
| N2 | 13 (18.1%) | 11 (15.3%) |  |
| N3 | 5 (6.94%) | 0 (0.00%) |  |
| NX | 0 (0.00%) | 1 (1.39%) |  |
| AJCC stage (M) |  |  | 1.000 |
| M0 | 63 (87.5%) | 62 (86.1%) |  |
| MX | 9 (12.5%) | 10 (13.9%) |  |
| Radiotherapy |  |  | 0.120 |
| No/Unknown | 68 (94.4%) | 72 (100%) |  |
| Yes | 4 (5.56%) | 0 (0.00%) |  |
| Chemo/endocrinotherapy |  |  | 0.013 |
| No/Unknown | 65 (90.3%) | 72 (100%) |  |
| Yes | 7 (9.72%) | 0 (0.00%) |  |

Supplementary Table 5, Clinical demographic status of high and low score groups in the TCGA cohort; Student’s t-test or Wilcoxon rank-sum test was used depending on the types of variables. Brackets indicate 25% and 75% quartiles.


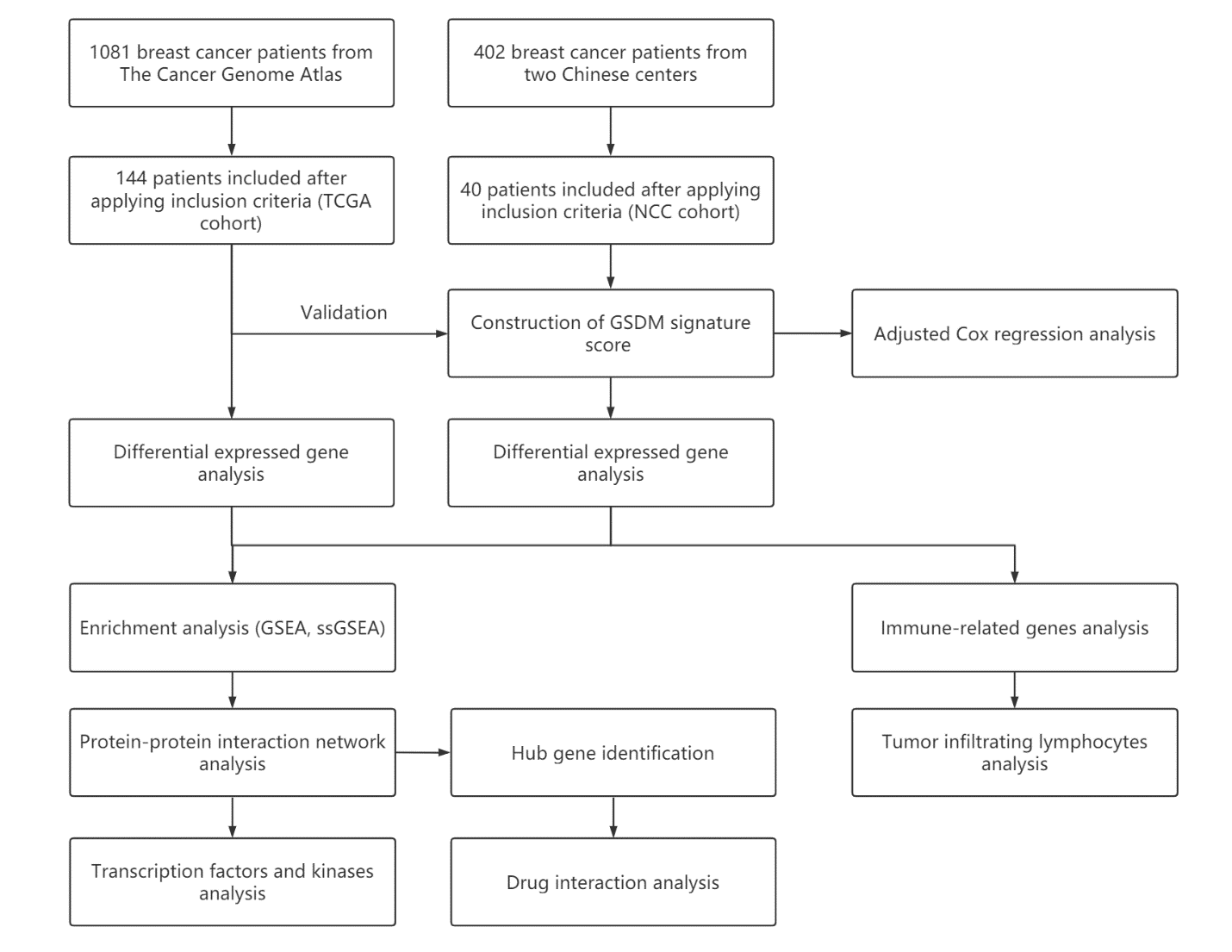


Supplementary Figure 1: Flow chart of the study design


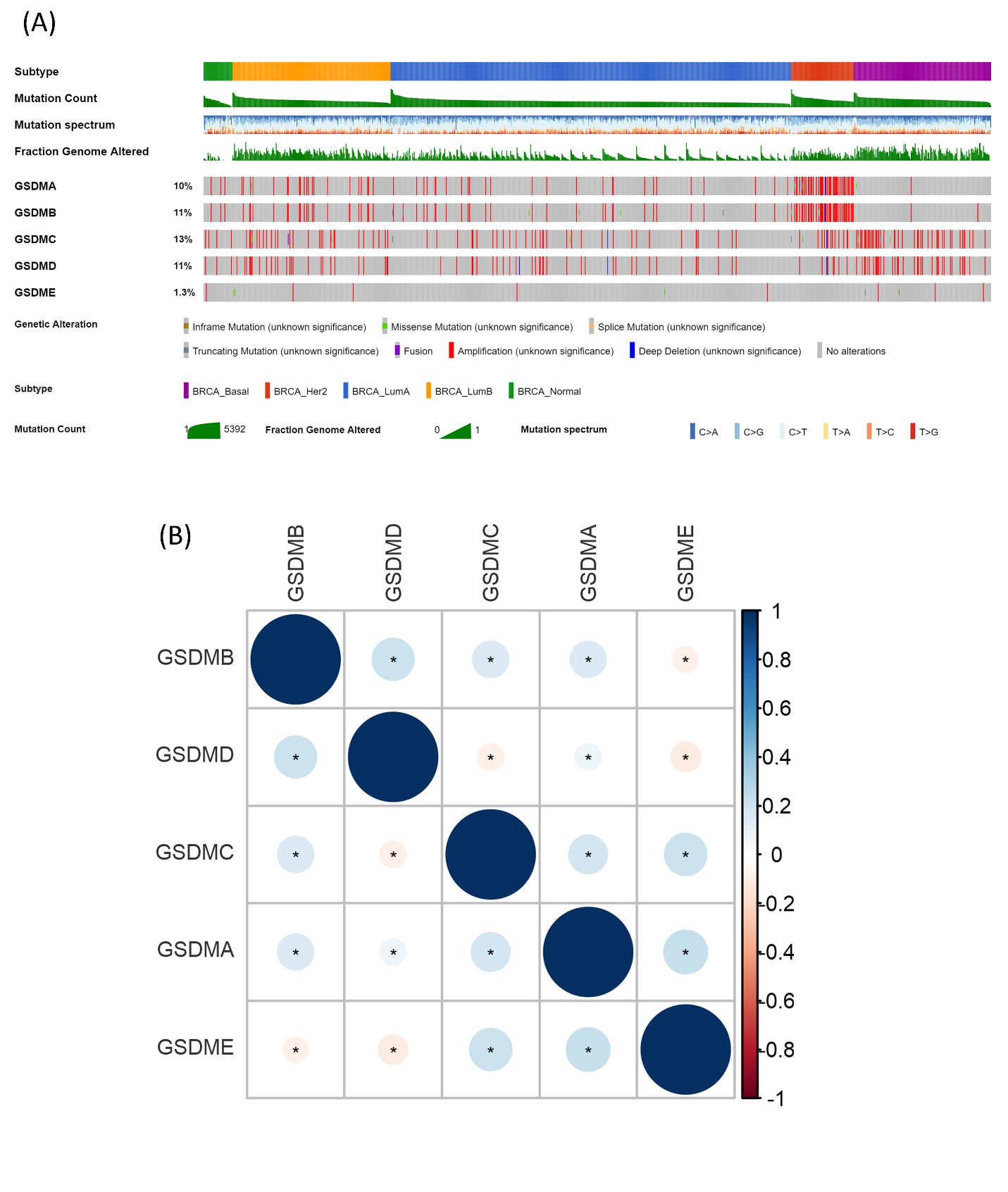


Supplementary Figure 2: **A**, Oncoprint plot of GSDM mutations in BRCA from the TCGA cohort (n = 1084). **B**, Correlation between different GSDMs in tumor tissues, colors represent values of correlation coefficients, * denotes significance.


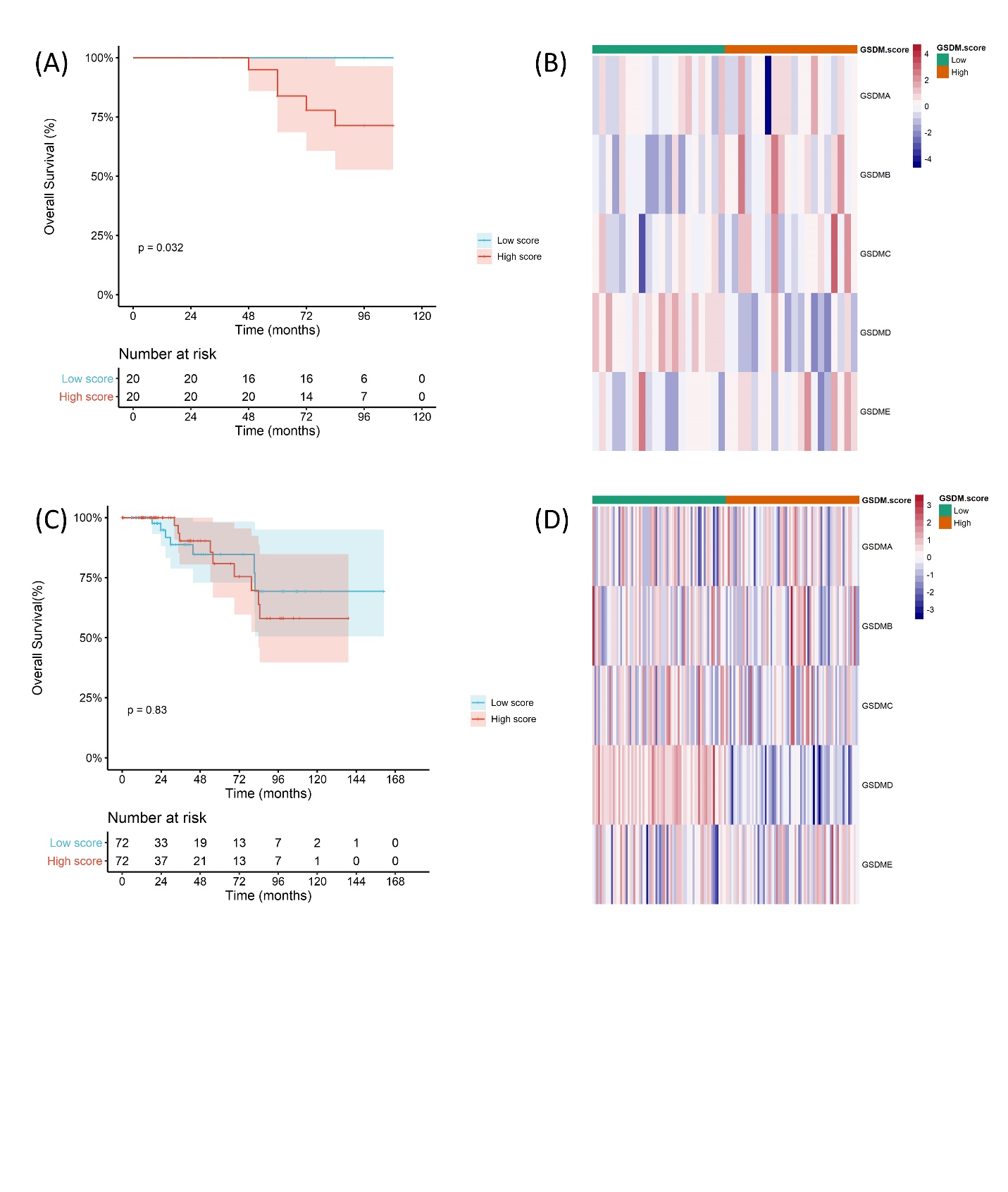


Supplementary Figure 3: **A**, Kaplan-Meier plot of OS in high GSDM score group (n = 20) compared with low GSDM score group (n = 20, p = 0.032). **B**, Kaplan-Meier plot of OS in high GSDM score group (n = 72) compared with low GSDM score group (n = 72, p = 0.83). **C**, Heatmap of Log2 transformed expression of GSDMs between the two groups. **D**, Heatmap of Log2 transformed expression of GSDMs between the two groups. A, C: NCC cohort; B, D: TCGA cohort.


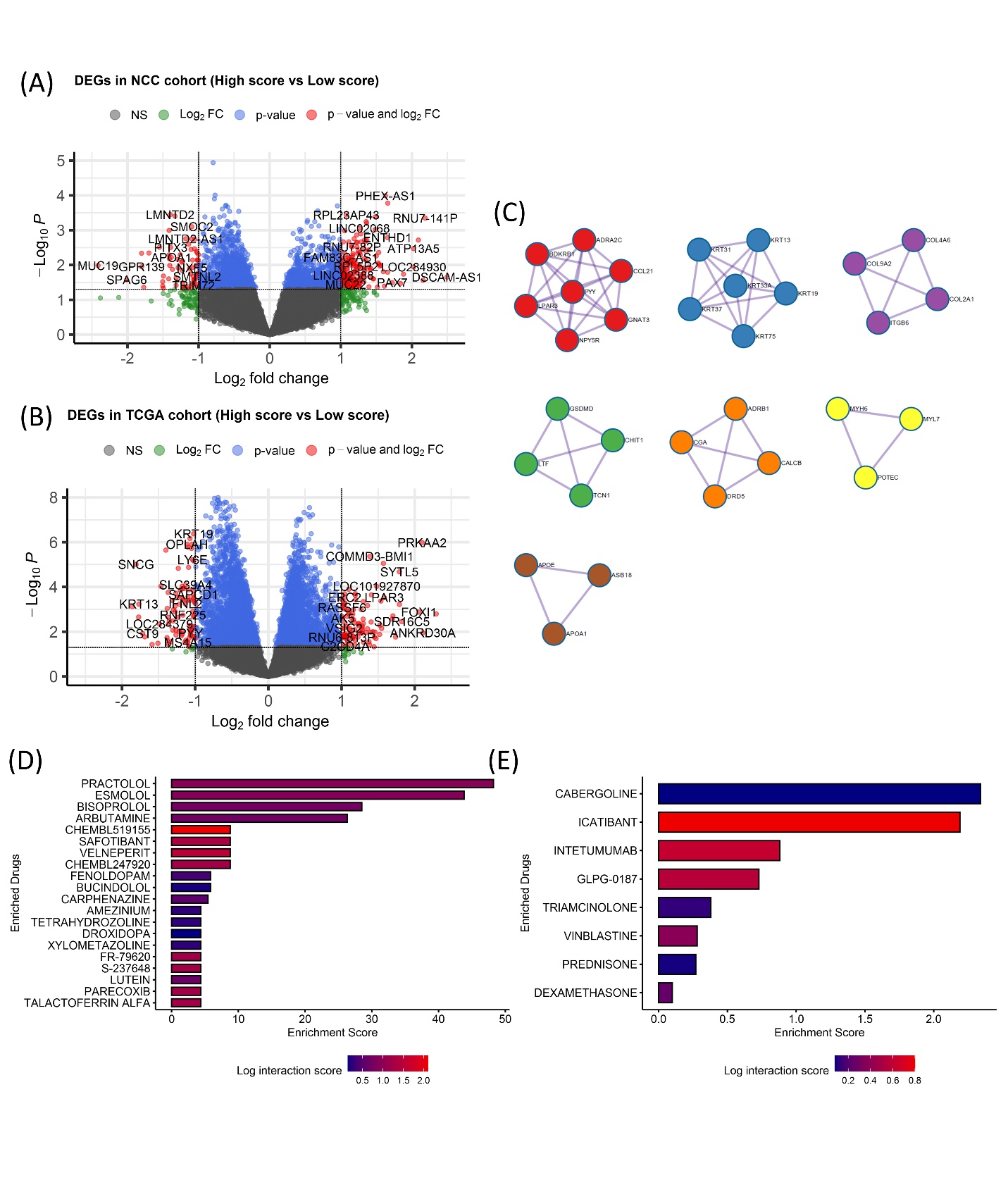


Supplementary Figure 4: **A**, Volcano plot of differentially expressed genes between high and low score groups in the NCC cohort, top differentially regulated genes were annotated. **B,** Volcano plot of differentially expressed genes between high and low score groups in the TCGA cohort, top differentially regulated genes were annotated. **C**, MCODE clusters of hub genes from the PPI network. **D**, Top interacting drugs and small chemicals with the hub genes. **E**, Top interacting FDA-approved anti-neoplastic drugs with the hub genes. All with FDR < 0.05 and p-values < 0.05.


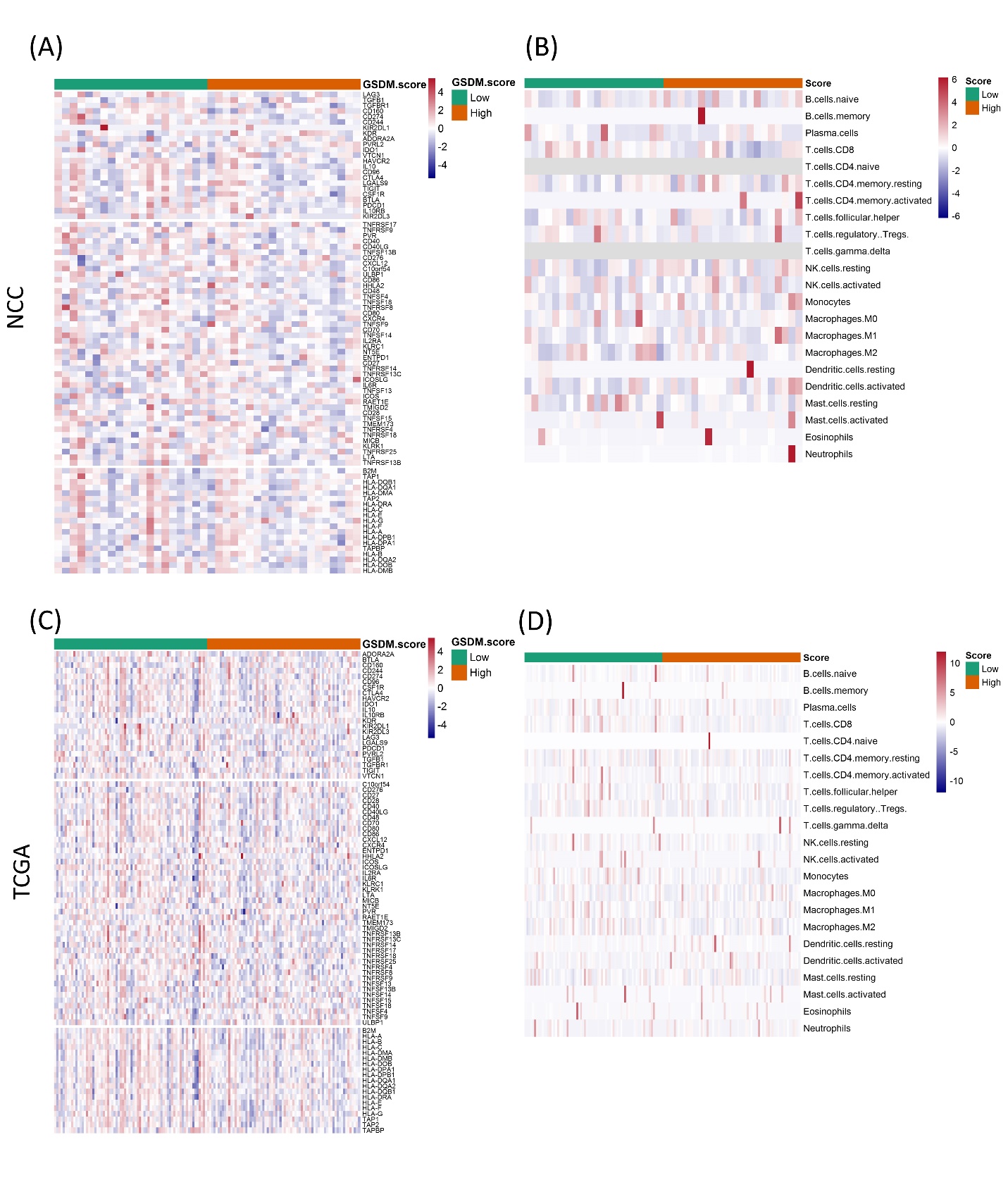


Supplementary Figure 5: **A**, Heatmap of immune-related genes (immunoinhibitors, immunostimulators, MHC molecules) between the high and low score groups; **B**, Heatmap of tumor infiltrating lymphocytes analyzed by CIBERSORTx between high and low score groups. **C**, Heatmap of immune-related genes between the high and low score groups. **D**, Heatmap of tumor infiltrating lymphocytes analyzed by CIBERSORTx between high and low score groups. A, B: NCC cohort; C, D: TCGA cohort.
